# Diurnal variation of the potassium layer in the upper atmosphere

**DOI:** 10.1002/2015GL063718

**Published:** 2015-05-15

**Authors:** W. Feng, J. Höffner, D. R. Marsh, M. P. Chipperfield, E. C. M. Dawkins, T. P. Viehl, J. M. C. Plane

**Affiliations:** ^1^ School of ChemistryUniversity of LeedsLeedsUK; ^2^ NCAS, School of Earth and EnvironmentUniversity of LeedsLeedsUK; ^3^ Leibniz‐Institute of Atmospheric PhysicsUniversity of RostockKühlungsbornGermany; ^4^ National Center for Atmospheric ResearchBoulderColoradoUSA

**Keywords:** WACCM‐K, lidar, diurnal variation

## Abstract

Measurements of the diurnal cycle of potassium (K) atoms between 80 and 110 km have been made during October (for the years 2004–2011) using a Doppler lidar at Kühlungsborn, Germany (54.1°N, 11.7°E). A pronounced diurnal variation is observed in the K number density, which is explored by using a detailed description of the neutral and ionized chemistry of K in a three‐dimensional chemistry climate model. The model captures both the amplitude and phase of the diurnal and semidiurnal variability of the layer, although the peak diurnal amplitude around 90 km is overestimated. The model shows that the total potassium density (≈ K + K^+^ + KHCO_3_) exhibits little diurnal variation at each altitude, and the diurnal variations are largely driven by photochemical conversion between these reservoir species. In contrast, tidally driven vertical transport has a small effect at this midlatitude location, and diurnal fluctuations in temperature are of little significance because they are small and the chemistry of K is relatively temperature independent.

## Key Points

- Pronounced local time dependency in the K number density
- WACCM‐K simulates the diurnal variation of K in good agreement with observation
- Photochemistry plays an important role in the diurnal variation of K

## 1 Introduction

Layers of metal atoms in the upper mesosphere and lower thermosphere (MLT) region have been observed by twilight photometry and lidar since the mid‐1960s [*Plane*, 1991]. The metals are produced by meteoric ablation. Observations of the layers, combined with laboratory studies of reaction rate coefficients and modeling, have contributed significantly to our understanding of chemical and dynamical processes in the MLT [*Plane*, 2003; *Plane et al.,* 2015]. The metal layers are affected by transport processes ranging from the hemispheric‐scale meridional circulation to small‐scale perturbations from gravity waves and tides [*Eska and Höffner*, 1998; *Feng et al.,* 2013; *Lübken et al.,* 2011; *Marsh et al.,* 2013]. A recent development has been the inclusion of the chemistry of Na [*Marsh et al.,* 2013], Fe [*Feng et al.,* 2013], K [*Plane et al.,* 2014], and Mg [*Langowski et al.,* 2015] in a three‐dimensional (3‐D) whole atmosphere model.

Atmospheric tides have an important impact in the upper atmosphere. Much work has been conducted on investigating the MLT tidal signature using satellite, ground‐based measurements and models, with a primary focus on the temperature and wind components [e.g., see *Ward et al.,* 2010] as well as atmospheric constituents [e.g., *Marsh and Roble,* 2002; *Smith et al.,* 2010]. The first study of potential tidal impacts on the Na layer density was conducted by *Clemesha et al.* [1982], who found that there was no significant diurnal variation in the Na density, which suggested that the tide had little impact on the Na layer at 23°S. In contrast, *Zhou et al.* [2008], *Yu et al.* [2012], and *Lübken et al.* [2011] have all reported significant diurnal variations of the Fe layer, which indicate a combination of tidal and photochemical influences. The response of K is of particular interest, as the K layer exhibits a completely different seasonal behavior to the other observable metals; a semiannual seasonality (summer and winter maxima) versus the annual variation (winter maximum and summer minimum) seen in other metals like Na and Fe [*Plane et al.,* 2014]. As yet, there have been no direct comparisons of the observed and modeled response of the metals layers to the atmospheric solar tide and photochemistry in the MLT.

In this paper, we use a combination of lidar measurements and modeling to investigate the impact of chemistry and dynamics on the diurnal behavior of the K layer. The paper is organized as follows: Section 2 describes the measurements and model used in the study. Section 3 compares the model results with lidar observations by examining important parameters of the K layer as well as diagnosing the diurnal and semidiurnal amplitude and phase of the K density variation. The model results are then used to compare the role of photochemistry with tidally driven transport and temperature fluctuations. Section 4 summarizes the conclusions.

## 2 Potassium Doppler Lidar Measurements and the WACCM‐K Model

Measurements of the K(D_1_) resonance wavelength at 770 nm [see *von Zahn and Höffner,* 1996; *Höffner and Lübken,* 2007] have been made at Kühlungsborn (54.1°N, 11.7°E) across the period 2002 to 2012. Here we use measurements in October averaged from 2004 to 2011, because this provides the longest available monthly observational record of K with a total of 363 h on 18 days of measurement, a sufficiently long data set to investigate the diurnal variation of K. The large number of available days from different years allows the averaged diurnal variability, rather than a snapshot from a few consecutive days, to be determined. After stacking all data into a single day with 1 h resolution, gravity waves and other disturbances are mostly averaged out by the large number of long measurements. Whereas K densities can be measured over a 24 h diurnal cycle, this is often not possible for temperatures because the temperature analysis requires a much better signal to noise. Temperatures are derived from the Doppler broadening of the K line, which is a relatively small effect. The large solar background during daytime, when exacerbated by the haze which often occurs over Kühlungsborn at noon, does not allow the accurate determination of temperatures. As a result, only a few temperatures with relatively large uncertainties are available around midday. It is possible to determinate temperature tides if shorter measurements are considered. For measurements longer than 6 h a tidal analysis has been performed by *Kopp et al.* [2014] for a different data set. A tidal analysis of temperature variations would therefore contain data from a small subset of the days that have been used for the K density analysis, and so this is not included in the present study. We also note that *Lübken et al.* [2011] have reported a comparison of the temperature and Fe density tidal variations using a mobile Fe lidar, but this was possible because of the nearly solar background free measurements under all conditions which cannot be achieved with a K or Na Doppler lidar.

A global model of meteoric K (WACCM‐K) has been developed recently [*Plane et al.,* 2014]. WACCM‐K combines three components: the Whole Atmosphere Community Climate Model (WACCM) which includes the important MLT chemistry and dynamical features [*Marsh et al.,* 2013]; a description of the neutral and ion‐molecule chemistry of K in the MLT; and the injection rate profile of K atoms which is calculated by combining a meteoric ablation model with an astronomical model of near‐Earth cosmic dust. The ablation flux of K during October at the latitude of Kühlungsborn is 150 atom cm^−2^ s^−1^. WACCM‐K successfully reproduces the observed semiannual variation in the K layer [*Plane et al.,* 2014]. WACCM also reproduces diurnal and semidiurnal tide in the MLT [*Chang et al.,* 2012]. Here we have used the same specified dynamics version nudged with the Goddard Earth Observing System 5 (GEOS‐5) meteorological data set [*Feng et al.,* 2013; *Langowski et al.,* 2015; *Plane et al.,* 2014], with a horizontal resolution of 1.9° × 2.5° and ~3.5 km vertical resolution in the MLT. The Prandtl number is set to 4. We sampled the model output every 30 min for Kühlungsborn from January 2004 until the end of December 2011.

A linear least squares fit method is also used to diagnose the diurnal/semidiurnal amplitude and phase of K layer, and the modeled temperature (*T*) and vertical wind velocity (*w*):

(1) $$f\left(t\right)=f_{0}+\sum_{n}A_{n}\cos\left(2n\pi t/24+\phi_{n}\right)$$

where *f*(*t*) is the time series of K, *T*, or *w.* The local time is *t*, *f*_0_ is diurnal mean value, *n* is wave number where *n* = 1 describes the diurnal variation while *n* = 2 describes the semidiurnal variation. *A_n_* is amplitude and *ϕ*_*n*_ is phase.

The K chemical lifetime (*τ*) with respect to conversion to the reservoirs KHCO_3_ and K^+^ can be derived from the chemical reaction set listed in Table 1:

(2) $$\begin{array}{c} \tau^{-1}=\left[k_{1}\left[O_{3}\right]+k_{3}\left[O_{2}\right]\left[M\right]\left(\frac{k_{4}\left[O\right]}{k_{4}\left[O\right]+J_{11}}\right)\right]\left[\frac{k_{5}\left[H_{2}O\right]+k_{6}\left[H_{2}\right]}{k_{2}\left[O\right]+k_{5}\left[H_{2}O\right]+k_{6}\left[H_{2}\right]}\right]\left[\frac{k_{8}\left[CO_{2}\right]\left[M\right]}{k_{8}\left[CO_{2}\right]\left[M\right]+k_{7}\left[H\right]+J_{12}}\right]\\ +k_{9}\left[NO^{+}\right]+k_{10}\left[{O_{2}}^{+}\right]+J_{13} \end{array}$$

**Table 1:** Neutral and Ionic Gas‐Phase Reactions Required to Estimate the Lifetime of K With Respect to Conversion to the Reservoir Species KHCO_3_ and K^+^

Number	Reaction	Rate Coefficient^a^
*Neutral Chemistry*		
R1	K + O_3_ → KO + O_2_	1.15 × 10^−9^ exp(−120/*T*)
R2	KO + O → K + O_2_	2 × 10^−10^ exp(−120/*T*)
R3	K + O_2_ (+ M) → KO_2_	1.3 × 10^−29^ (*T*/200)^−1.23^
R4	KO_2_ + O → KO + O_2_	2 × 10^−10^ exp(−120/*T*)
R5	KO + H_2_O → KOH + OH	2 × 10^−10^ exp(−120/*T*)
R6	KO + H_2_ → KOH + H	2 × 10^−10^ exp(−120/*T*)
R7	KOH + H → K + H_2_O	2 × 10^−10^ exp(−120/*T*)
R8	KOH + CO_2_ (+ M) → KHCO_3_	7.1 × 10^−28^ (*T*/200)^‐4.2^
*Ion‐Molecule Chemistry*		
R9	K + NO^+^ → K^+^ + NO	9.4 × 10^−10^
R10	K + O_2_^+^ → K^+^ + O_2_	3.2 × 10^−9^
*Photochemical Reactions*		
R11	KO_2_ + *hν* → K + O_2_	2.2 × 10^−3^
R12	KOH + *hν* → K + OH	2.7 × 10^−2^
R13	K + *hν* → K^+^ + *e* ^−^	4 × 10^−5^

a Rate coefficients from *Plane et al.* [2014]. Units: unimolecular, s^−1^; bimolecular, cm^3^ molecule^−1^ s^−1^; termolecular, cm^6^ molecule^−2^ s^−1^.

The first term on the right‐hand side describes the rate of conversion of K to KHCO_3_, with the short‐lived intermediates KO, KO_2_, and KOH assumed to be in chemical steady state; the final three terms define the rate of conversion to K^+^ via charge transfer and photoionization.

## 3 Results and Discussion

**Figure 1:**
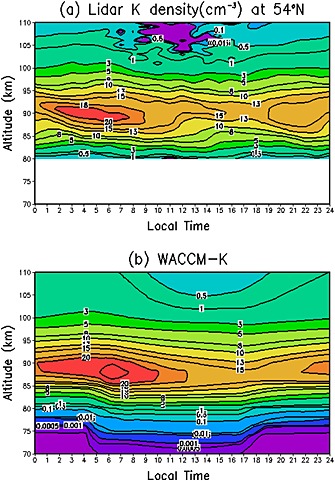
Diurnal variation of the K layer for October at Kühlungsborn, Germany (54°N, 12°E), averaged from 2004 to 2011. (a) K density (atom cm^−3^) as a function of altitude and local time, measured by lidar. (b) K density as a function of altitude and time from WACCM‐K.

Figure 1 shows the observed and modeled diurnal variation of the K layer. The measurements show that the K layer peaks around 90 km with a maximum density of ~20 cm^−3^ between 0300 and 0900 LT, which then decreases to ~13 cm^−3^ around 2100 LT (Figure 1a). Overall, WACCM‐K simulates the observed K layer quite well and also captures the large differences between dawn (around 0600 LT) and dusk (around 1800 LT) at 90 km (Figure 1b). The layer bottomside production and loss of K is similar to Fe [*Feng et al.,* 2013; *Yu et al.,* 2012]. There is a large decrease of observed neutral K around 105–110 km which is due to the conversion of K to K^+^ ions, through charge transfer with NO^+^ and O_2_^+^ and photoionization. This observed decrease in neutral K is also well captured by the model, although WACCM‐K overestimates the daytime atomic K density around 105–110 km by a factor of 5. As we have discussed in *Feng et al.* [2013], this likely arises because there is an accumulation in the lower thermosphere of K^+^ ions (and hence K, with which K^+^ is in quasi steady state) since WACCM does not include the Lorentz force which distributes long‐lived metal ions over greater heights in the thermosphere.

**Figure 2:**
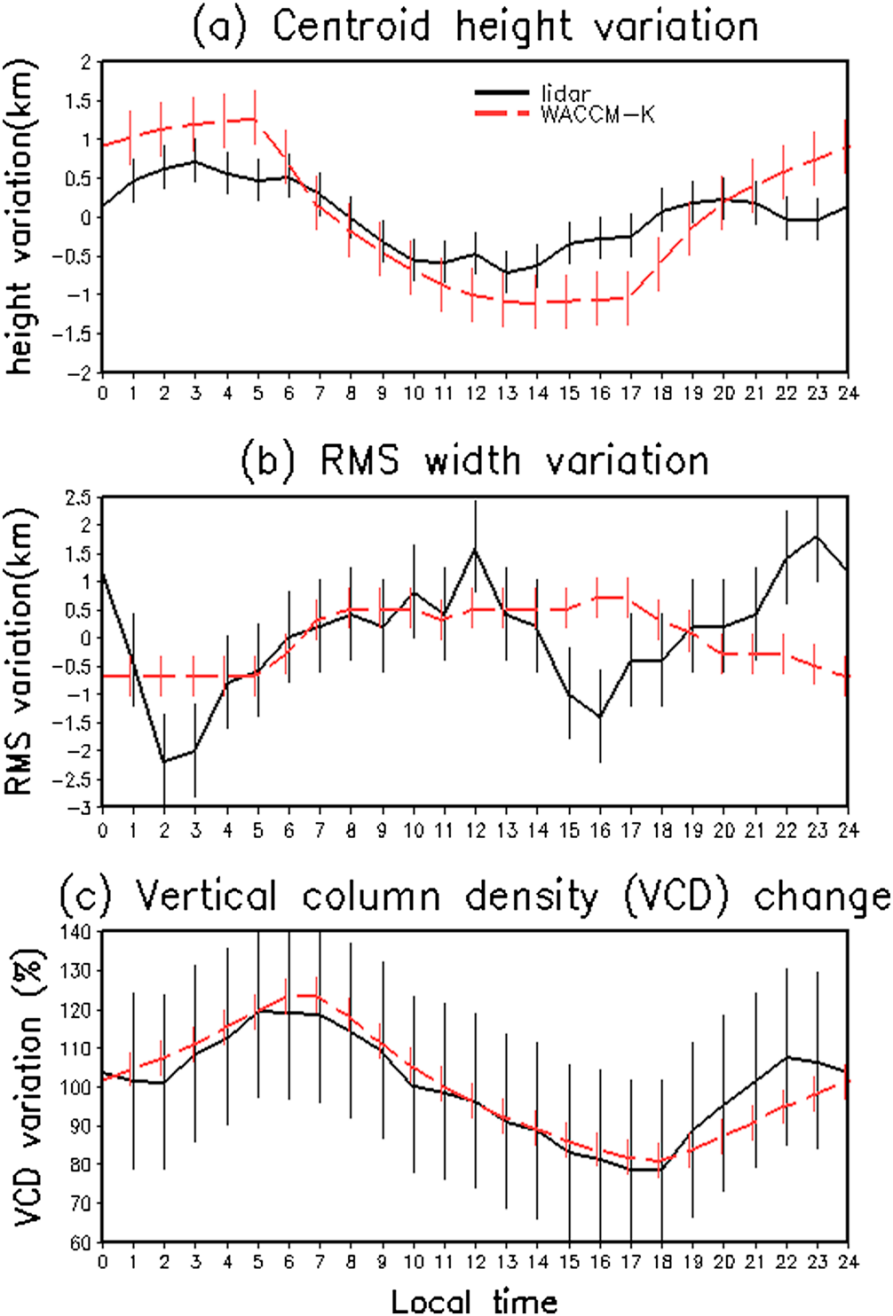
Diurnal variation of (a) the K centroid height (km), (b) RMS width (km) which are subtracted from the 24 h averaged value, and (c) the vertical column density (VCD) changes (average = 100%) over 24 h. The error bars are calculated from standard deviation of the October mean K density.

In order to explore the characteristics of the K layer in greater detail, we have calculated the centroid height and RMS (root‐mean‐square) width, which is a measure of the layer thickness, as well as the K column abundance. Figure 2 shows the diurnal variation of the K centroid height, RMS width minus its 24 h averaged value as well as K vertical column density (VCD) percentage change over 24 h (the average column density is 100%) calculated from the K density in Figure 1. This highlights any changes caused by tides which move the layer up/down within 24 h. WACCM‐K captures the mean K characteristics quite well. The mean K centroid height is 90.9 km from lidar while it is 90.2 km from WACCM‐K. The observed K RMS width is 4.7 km and the modeled width is 4.4 km, the observed K VCD (integrated from 80 to 110 km) is 1.99 × 10^7^ cm^−2^ and the modeled column density is 2.04 × 10^7^ cm^−2^. Both lidar and model show that the centroid height of K layer occurs at nighttime then decreases during the day. The width of the K layer from lidar varies from a minimum at 0200–0300 LT to a maximum at midnight, whereas the model somewhat overestimates and then underestimates it, though the mean K RMS width is very similar to that measured. Both model and observation show that the K column abundance increases from 1800 LT to a maximum around 0700 LT and then decreases throughout the daytime. Clearly, WACCM‐K reproduces the observed K column abundance variation quite well though the model slightly overestimates the total column abundance value.

**Figure 3:**
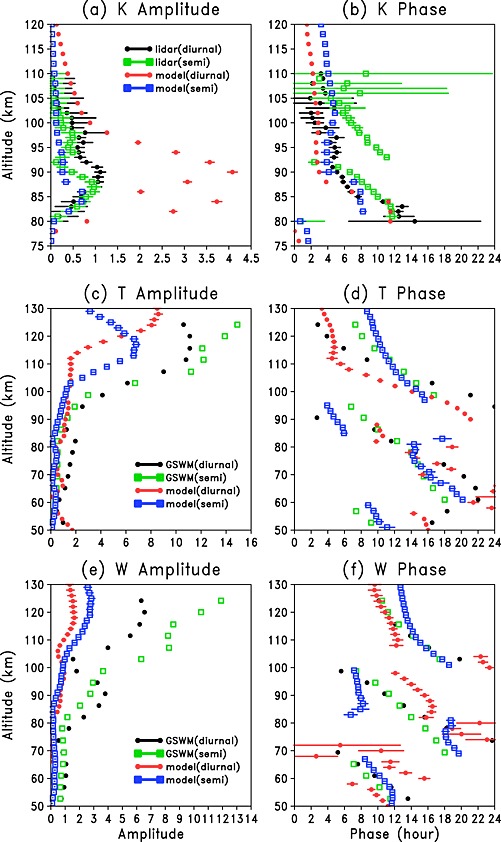
The diagnosed amplitude and phase (h) of the diurnal and semidiurnal variations in: (a and b) K (cm^−3^) from lidar and WACCM‐K model, (c and d) temperature, and (e and f) vertical wind (cm/s) as a function of altitude from GSWM and WACCM‐K model simulations. The error bars represent 1 sigma uncertainty from the regression calculation.

Figures 3a and 3b show height profiles of the amplitude and phase (diurnal and semidiurnal) for the measured and modeled K density. The observed maximum diurnal amplitude is 1.1 cm^−3^ around 90 km, while the maximum semidiurnal amplitude is 1.0 cm^−3^ around 87 km. Although the maximum diurnal and semidiurnal K amplitude from the model is at a similar altitude to that observed, the model largely overestimates the observed K diurnal amplitude maximum. Above 105 km, there is significant sharp decrease in the observed K diurnal amplitudes, whereas the model still exhibits large diurnal modeled K amplitude. This is due to the overestimation of the K density above 105 km by the model compared with lidar observation (Figure 1). The K diurnal and semidiurnal phase is shown in Figure 3b, which is defined with respect to the time of maximum K in the oscillation. At the layer peak altitude, the diurnal and semidiurnal variations peak near sunrise in both observations and the model. The observed diurnal K phase indicates the time of maximum K perturbation is ~ 14 h at 80 km down to 2 h around 110 km, which is well captured by the model. The model also correctly predicts a later semidiurnal phase than diurnal phase.

The potassium lidar temperature data are insufficient for detailed diurnal analysis. We have therefore compared the tidal components of the temperature *T* (Figures 3c and 3d) and vertical velocity *w* (Figures 3e and 3f) in WACCM‐K with those from the global scale wave model (GSWM) [*Hagan et al.,* 1995], which has been widely used to investigate tides in MLT. GSWM predicts that the tidal amplitude of *T* for October around Kühlungsborn (Figure 3c) increases with altitude, and the semidiurnal amplitude (with a maximum value of 14–16 K around 125 km) is larger than the diurnal temperature amplitude above 100 km, whereas WACCM underestimates both the diurnal and semidiurnal *T* amplitudes. However, below 100 km the models are in good agreement (less than 5 K difference). Generally, both the diurnal and semidiurnal phases for *T* from GSWM and WACCM (Figure 3d) exhibit downward progression between 60 and 90 km and above 95 km. There is upward progression for the diurnal *T* between 94 and 98 km and semidiurnal *T* between 90 and 95 km. *Kopp et al.* [2014] reported temperature phases and amplitudes for October using a Rayleigh‐Mie‐Raman (RMR) lidar. Even though this data set differs from the data set herein, it gives an indication of the observed temperature tide during the period discussed here. We note that WACCM and GSWM have lower diurnal and semidiurnal amplitudes (less than 3 K) between 85 and 95 km compared with *Kopp et al.* [2014] (1–8 K).

The amplitudes of the diurnal and semidiurnal tidal components of *w* from both GSWM and WACCM (Figure 3e) also increase with altitude in the MLT region. The semidiurnal variation in *w* dominates above 100 km. WACCM appears to capture the main features of the diurnal and semidiurnal tide both in amplitude and phase, though it has smaller amplitude compared with GSWM at this midlatitude location.

**Figure 4:**
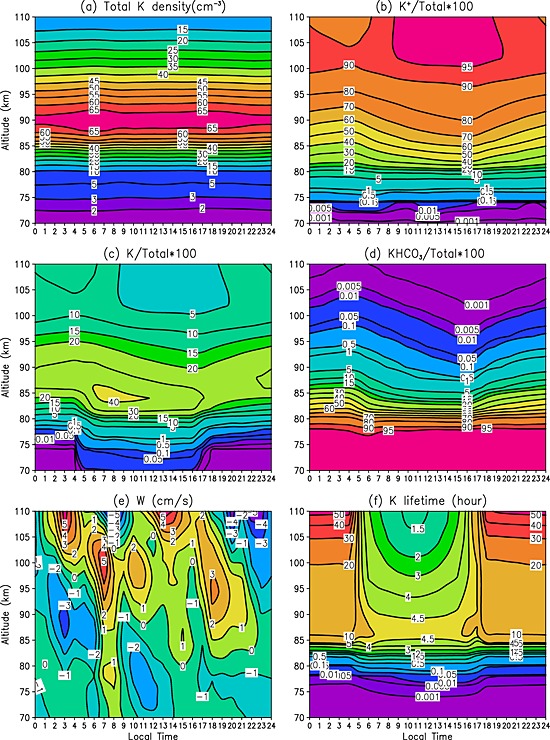
Diurnal variation of simulation of (a) the total K layer, (b) the percentage of ionic K, (c) neutral K atoms and (d) reservoir species KHCO_3_ with respect to (e) the total K density and diagnosed vertical velocity and (f) chemical K lifetime for October at Kühlungsborn, Germany (54°N, 12°E), averaged from 2004 to 2011.

In order to investigate the possible effect of the tide on the diurnal cycle of the K layer, we start by considering how the total density of K‐bearing chemical species (which is essentially the sum of K, K^+^, and KHCO_3_ in WACCM‐K) varies as a function of height and local time. Figure 4a shows that the total K density peaks around 90 km (~65 cm^−3^), which is slightly higher than the peak altitude of neutral K atom density (Figure 1b). Significantly, the total K is almost constant at each height over a diurnal cycle, which suggests that vertical transport plays a minor role. Figures 4b–4d show the vertical profiles as a function of time of the three main species K^+^, K, and KHCO_3_ expressed as a percentage fraction of total K. K^+^ ions dominate on the topside of the K layer, accounting for more than 60% of total K above 90 km and more than 90% above 100 km. Neutral K atoms comprise 20–40% of the total density around the layer peak, with very small insignificant contributions below 80 km and above 100 km. KHCO_3_ is the dominant reservoir species on the bottomside of the K layer, contributing more than 90% below 79 km. Other K‐containing species make very small contributions [*Plane et al.,* 2014].

Above 87 km, K and K^+^ simply interconvert over a diurnal cycle: K is converted into K^+^ during daytime through photoionization and charge exchange reactions, and K^+^ is neutralized through cluster formation and dissociative recombination with electrons [*Plane et al.,* 2014]. Photochemistry also explains the buildup of neutral K atoms below 87 km after sunrise via the photolysis of the reservoir species KHCO_3_. The marked increase in K between 83 and 87 km after ~0600 LT disappears again by 1200 LT, due to conversion of atomic K into K^+^. On the underside of the layer below 85 km, there is a complete removal of K during nighttime (1800–0500 LT), as K is converted into the dominant reservoir KHCO_3_. After dawn this reservoir is converted back to K by photolysis. Furthermore, the conversion of K back to KHCO_3_ involves three steps: the reaction of K with O_3_ to form KO; reaction of KO with H_2_O/H_2_ to yield KOH; and recombination of KOH with CO_2_ [*Plane et al.,* 2014]. The rates of these three steps in the mechanism slow down after sunrise. This is because during daytime at 74 km more than 70% of the O_3_ is photolyzed, and the daytime O and H concentrations are ~50 and 10 times higher than during the night, respectively (these variations are illustrated in Figure 4 of *Plane et al.* [2015]). Thus, during the day K is oxidized more slowly by O_3_, and the increased O and H radical concentrations convert KO and KOH, respectively, more rapidly back to K.

The diurnal variation in temperature predicted by WACCM is small over Kühlungsborn: less than 4 K at each height between 80 and 100 km, compared with RMR measurements between 85 and 95 km [*Kopp et al.,* 2014]. Since the temperature dependencies of the relevant reactions in the K chemistry scheme are comparatively small (and the reaction of KHCO_3_ + H has too large an activation energy to be important) [*Plane et al.,* 2014], diurnal variations in temperature do not play a significant role in the diurnal variation of the K layer. Figure 4e shows that the modeled vertical wind *w* at 54°N during October is small (≤5 cm s^−1^) in the MLT, corresponding to tidally driven vertical displacements of less than 1 km. This lack of vertical transport explains the near‐constant total K density at each height (Figure 4a).

Finally, Figure 4f shows the K lifetime *τ* calculated using equation (2). Below 80 km, *τ* is short (<10 min), although it effectively doubles at each height during the day because of the increased concentrations of O and H and decreased O_3_ (see above). The longest lifetimes are during the night, less than 1 day below 100 km, and much longer higher in the thermosphere. However, during daytime the lifetime is always less than 4.5 h throughout the MLT. This explains why in situ photochemistry plays a much more important role than vertical transport in controlling the diurnal variation of atomic K.

## 4 Conclusions

The diurnal variation of the midlatitude K layer has been measured using a lidar located at Kühlungsborn (54.1°N, 11.7°E), Germany. Data obtained between 2004 and 2011 were used to construct a height profile of the K density as a function of local time, for the month of October. Even though this is a single site observation, the data provide a comprehensive test for the interplay between dynamics and chemistry in a 3‐D model of the K layer. Overall, WACCM‐K reproduces the observed diurnal and semidiurnal variations in the K layer satisfactorily. It is shown that most of the variability is accounted for by photochemistry, both directly through photolysis of the reservoir species KHCO_3_ and photoionization of K and indirectly by changing the concentrations of O_3_, O, and H which affect the rates of important reactions. Diurnal variations in temperature, and tidally driven vertical transport, appear to play a minor role in the upper atmospheric potassium layer at this midlatitude location.
